# Polymorphisms in *CALCRL* and *MPC1* Genes and Their Associations with Growth Traits in Tianzhu White Yaks

**DOI:** 10.3390/ani16182944

**Published:** 2026-09-19

**Authors:** Tian Gu, Xuedong Qi, Yongfu La, Xiaoming Ma, Wenxue Luo, Guowu Yang, Wenwen Ren, Zhenyu Zhang, Min Chu, Xiaoyun Wu, Xian Guo, Wanzhen Qi, Chunnian Liang

**Affiliations:** 1 Key Laboratory of Yak Breeding Engineering of Gansu Province, Lanzhou Institute of Husbandry and Pharmaceutical Sciences, Chinese Academy of Agricultural Sciences, Lanzhou 730046, China; gutian20011121@163.com (T.G.); layongfu@caas.cn (Y.L.); maxiaoming@caas.cn (X.M.); 821012410409@caas.cn (G.Y.); rww15806106435@126.com (W.R.); zzy960911@163.com (Z.Z.); chumin@caas.cn (M.C.); wuxiaoyun@caas.cn (X.W.); guoxian@caas.cn (X.G.); 2 Key Laboratory of Animal Genetics and Breeding on Tibetan Plateau, Ministry of Agriculture and Rural Affairs, Lanzhou 730050, China; 3 College of Animal Science and Veterinary Medicine, Southwest Minzu University, Chengdu 610041, China; 4 Tianzhu Autonomous County Animal Husbandry Technology Extension Station, Tianzhu 733200, China; tzxqxd@163.com (X.Q.); tzxlwx@163.com (W.L.)

**Keywords:** Tianzhu white yak, *CALCRL* gene, *MPC1* gene, SNP, body weight, body measurements

## Abstract

Single nucleotide polymorphisms (SNPs) may contribute to variation in economically important body traits in yak, but variants near calcitonin receptor-like receptor (*CALCRL*) and mitochondrial pyruvate carrier 1 (*MPC1*) have not been characterized in Tianzhu white yak. We evaluated two downstream SNPs in 536 yaks (351 females and 185 males; 2–6 years old) using polymerase chain reaction amplification and Sanger sequencing. Associations with body height, body oblique length, chest circumference, and body weight were analyzed separately using general linear models adjusted for sex and age. The *CALCRL* gene chr 2:140178268 A > T locus was associated with body weight (*p* = 0.001). Individuals with AA and AT genotypes had significantly greater body weight than individuals with the TT genotype. The *MPC1* gene chr 10:98419837 C > T locus was associated with body height (*p* = 0.002), chest circumference (*p* < 0.001), and body weight (*p* < 0.001), but not body oblique length (*p* = 0.072); CC individuals generally showed greater values than TT individuals. Both SNPs are downstream non-coding variants. These loci are candidates for further investigation, but independent population validation and functional studies are required before their potential use in breeding programs.

## Simple Summary

Tianzhu white yaks are multipurpose livestock that support pastoral communities on the Qinghai–Tibet Plateau, where animals must cope with cold, low oxygen, and seasonal feed shortages. Genetic variants associated with body weight and body size may help improve genetic evaluation. In this study, 536 Tianzhu white yaks were examined for two candidate genetic variants. Body weight, body height, body oblique length, and chest circumference were compared among animals carrying different forms of each variant after accounting for age and sex. One variant was associated with body weight, whereas the other was associated with body height, chest circumference, and body weight. Because the measurements were collected at a single time point, the results describe differences in body weight and body size rather than growth rate. In addition, these two candidate loci were identified in the same research population, so they still need to be further verified in the independent yak population. These two loci may therefore serve as candidates for subsequent genetic and functional studies, but their potential use in breeding requires further validation.

## 1. Introduction

Tianzhu white yak (*Bos grunniens*) is an important indigenous livestock resource on the Qinghai–Tibet Plateau in China. It is famous for its pure white coat and is known as “White Pearl of Grassland” and “Qilian Snow Peony”. It is mainly distributed in the alpine grassland at elevations above 2500 m in Tianzhu Tibetan Autonomous County, Gansu Province. It is a local yak population shaped by long-term natural selection and artificial breeding [1]. Tianzhu White Yak is characterized by its distinctive white coat, which provides multiple products and services, including meat, milk, fiber, and other resources, and is an important livestock resource for local pastoral communities. However, the Tianzhu white yak has long been raised under cold and hypoxic plateau conditions, where the dry season is about half a year, and it generally exhibits relatively low body weight and small body size, and its milk and meat production performance is also lower than other yaks and cattle [2]. Studies have shown that the Qinghai–Tibet Plateau yak has the characteristics of low body weight, slow growth rate and early growth inflection point [3]. Therefore, body weight and body size are important traits to consider in breeding programs of Tianzhu white yak.

Body measurements are important phenotypic indicators in the genetic improvement of large livestock [4]. Body weight and body size are influenced by both genetic and environmental factors. With the development of genomic-assisted breeding and the improvement of genomic selection efficiency, it will be possible to protect and develop the breeding resources of Tianzhu white yak, thus promoting the modernization and industrialization of the Tianzhu white yak industry. Single nucleotide polymorphisms (SNPs) are abundant and relatively stable forms of variation in the genome, and have been widely used to study the genetic basis of important economic traits in livestock. Several SNP association studies have previously been reported in yak. For example, in a study of 409 Chinese indigenous yaks, Gui et al. identified two SNPs in the *SIRT1* and *H-FABP* genes by DNA sequencing, and both variants were associated with body weight and body length [5]. With the advancement in genome-wide studies, the scope of research on candidate markers associated with growth traits in yak has expanded substantially. A genome-wide association study in Maiwa yaks identified seven marker loci significantly associated with body weight and proposed *MFSD4*, *LRRC37B*, and *NCAM2* as candidate genes [6]. A multi-population study involving 31 yak breeds and genetic resources identified six significant markers associated with body height and further highlighted *FXYD6*, *SOHLH2*, *ADGRB2*, and *OSBPL6* as candidate genes [7]. In Ashidan yaks, SNPs in the *TXK* and *PLCE1* genes have been reported to be associated with body measurement traits [8]. In addition, whole-genome resequencing of Gannan yaks identified multiple body-weight-related genes and prioritized *DRC1* and S*ELENOI* as candidate genes [9]. These studies show that the genetic architecture of yak body size is polygenic and that candidate markers may differ among breeds, ages, and production environments.

The calcitonin receptor-like receptor gene (*CALCRL*) encodes a G protein-coupled receptor. The *CALCRL*-encoded receptor interacts with receptor activity-modifying proteins (RAMPs) to form receptor complexes with distinct functions that participate in physiological processes including angiogenesis, lymphangiogenesis, vascular regulation, cell proliferation, and apoptosis [10,11,12]. These functions provide a biological rationale for investigating *CALCRL* in relation to body-development phenotypes, although direct evidence linking *CALCRL* polymorphisms with body weight or body measurements in yak remains limited.

Mitochondrial pyruvate carrier 1 (*MPC1*) is a core component of the mitochondrial pyruvate carrier complex. It participates in the transport of pyruvate from the cytoplasm into the mitochondrial matrix, thereby linking glycolysis with the tricarboxylic acid (TCA) cycle [13,14]. Previous studies have implicated *MPC1* in cellular energy metabolism, skeletal-muscle metabolism, muscle-fiber development, and maintenance of systemic metabolic homeostasis [15,16,17]. These biological functions suggest that genetic variation near *MPC1* may be relevant to phenotypic variation in body weight and body dimensions.

*CALCRL*-mediated neuropeptide signaling is involved in osteoblast proliferation and somatic cell development, while *MPC1* dependent mitochondrial pyruvate transport is crucial for energy supply of skeletal muscle cells, which provide a biological rationale for investigating their relationships with the body weight and body size of animals. However, to our knowledge, associations between variants *CALCRL* or *MPC1* and body-weight or body-size phenotypes have not been reported in Tianzhu white yak.

The objective of this study was to investigate the associations of genotypes at two candidate loci, *CALCRL* gene chr 2:140178268 A > T and *MPC1* gene chr 10:98419837 C > T, with body weight, body height, body oblique length, and chest circumference in 536 adult Tianzhu white yaks. We hypothesized that genotypes at these two loci would be associated with variation in one or more body-weight or body-size phenotypes. The study was intended to provide candidate loci for subsequent validation and functional investigation.

## 2. Materials and Methods

### 2.1. Experimental Animals and Body Measurements

A total of 536 healthy Tianzhu white yaks, including 351 females and 185 males, were randomly selected from a single pastoral herd in Tianzhu Tibetan Autonomous County, Wuwei City, Gansu Province, China. The animals ranged from 2 to 6 years of age and were managed under the same local production system, consisting of natural grazing without concentrate supplementation. The average altitude of the test area is about 2500 m. The body weight (BW), body height (BH), body oblique length (BOL) and chest circumference (CC) of the experimental yak were measured in early November 2025. All the animals fasted for 12 h before phenotypic measurement, and all the individuals completed the above four phenotypic measurements according to the standardized measurement protocol.

Blood was collected from the jugular vein of the experimental animals and placed into vacuum blood-collection tubes containing anticoagulant. After collection, the samples were gently mixed, transported to the laboratory and stored at −20°C for subsequent genomic DNA extraction. All the test operations involving animals were carried out in accordance with the relevant provisions of the China Laboratory Animal Management and the Ministry of Agriculture and Rural Areas of the People’s Republic of China, and were approved by the Animal Management and Use Committee of Lanzhou Institute of Husbandry and Pharmaceutical Sciences, Chinese Academy of Agricultural Sciences (Permit No. 2025-51, 20 April 2025).

### 2.2. DNA Extraction

Genomic DNA was extracted from whole-blood samples using a TIANamp Genomic DNA Kit (TIANGEN Biotech (Beijing) Co., Ltd., Beijing, China). DNA concentration and quality were assessed using a NanoDrop™ 2000 spectrophotometer (Thermo Fisher Scientific, Waltham, MA, USA) and 1.5% agarose gel electrophoresis. DNA samples with A260/A280 ratios between 1.8 and 2.0 were retained for subsequent PCR amplification.

### 2.3. Candidate SNP Selection and Primer Design

The two candidate SNP loci analyzed in this study were *CALCRL* gene chr 2:140178268 A > T and *MPC1* gene chr 10:98419837 C > T. These two loci come from the genome-wide association analysis carried out by our research group in the early stage, and the age and sex factors have been corrected in the process of candidate locus screening. The genome-wide analysis represents a separate component of the broader research project and will be reported elsewhere.

In this study, the two candidate loci were subsequently genotyped. Referring to the European Nucleotide Archive of the Bosgu_v3.0 version of the yak genome, the upstream and downstream reference sequences of each locus were obtained and the corresponding FASTA files were downloaded. The specific primers were designed by using NCBI Primer-BLAST (https://www.ncbi.nlm.nih.gov/tools/primer-blast/, accessed on 14 September 2026), and the primers were synthesized by Sangon Biotech (Shanghai) Co., Ltd. (Shanghai, China). The sequence information and annealing temperature of each primer are shown in Table 1.

### 2.4. PCR Amplification and Sequencing

The selected SNP loci were amplified by PCR. The blood DNA of Tianzhu white yak was used as the template for PCR amplification. The total reaction volume was 20 μL, containing 8 μL ddH_2_O, 1 μL genomic DNA, 0.5 μL each of the forward and reverse primers, and 10 μL Premix Taq™ (TaKaRa Taq™ Version 2.0 plus dye; Takara Bio Inc., Kusatsu, Shiga, Japan). The PCR program consisted of: initial denaturation at 94 °C for 3 min; 35 cycles (denaturation at 94°C for 30 s, annealing at 59°C for 30 s, extension at 72°C for 20 s); final extension at 72°C for 5 min; followed by holding at 4 °C. The amplified products were detected by 1% agarose gel electrophoresis (voltage 110 V, electrophoresis for 35 min), and the amplified bands of the expected sizes were observed. The PCR products together with forward and reverse primers were sent to Sangon Biotech (Shanghai) Co., Ltd. (Shanghai, China) for Sanger sequencing.

### 2.5. Statistical Analysis

MEGA 12.0 was used to inspect the Sanger sequencing results and determine genotypes, and Microsoft Excel 2019 was used for data organization. Genotype and allele frequencies, expected heterozygosity (He), effective number of alleles (Ne), and polymorphism information content (PIC) were calculated, and Hardy–Weinberg equilibrium was evaluated for each locus.

The associations between each candidate SNP and the four phenotypic traits were analyzed separately using the general linear model (GLM) procedure in SPSS Statistics v26.0. Because age and sex may influence body weight and body measurements and could confound genotype comparisons, both factors were included in the association model. Sex was treated as a fixed effect and age was included as a continuous covariate. For each SNP and phenotype, the following model was used:

$$Y_{n}=\mu +G_{i}+S_{j}+\beta A_{n}+e_{n}$$

Among them, *Y_n_* is the phenotypic value of the nth animal for body weight, body height, body oblique length, or chest circumference; *μ* is the overall mean; *G_i_* is the fixed effect of SNP genotype; *S_j_* is the fixed effect of sex; *A_n_* is the age of the nth animal; *β* is the regression coefficient for age; and *e_n_* is the residual error.

Genotype-specific phenotypic values are reported as estimated marginal means ± standard errors (SE), adjusted for age and sex. Pairwise comparisons among genotype classes were performed using the Bonferroni adjustment. Differences were considered statistically significant at *p* < 0.05.

## 3. Results

### 3.1. Genotyping and Genetic Diversity Parameters of the CALCRL and MPC1 Linked SNPs

**Figure 1:**
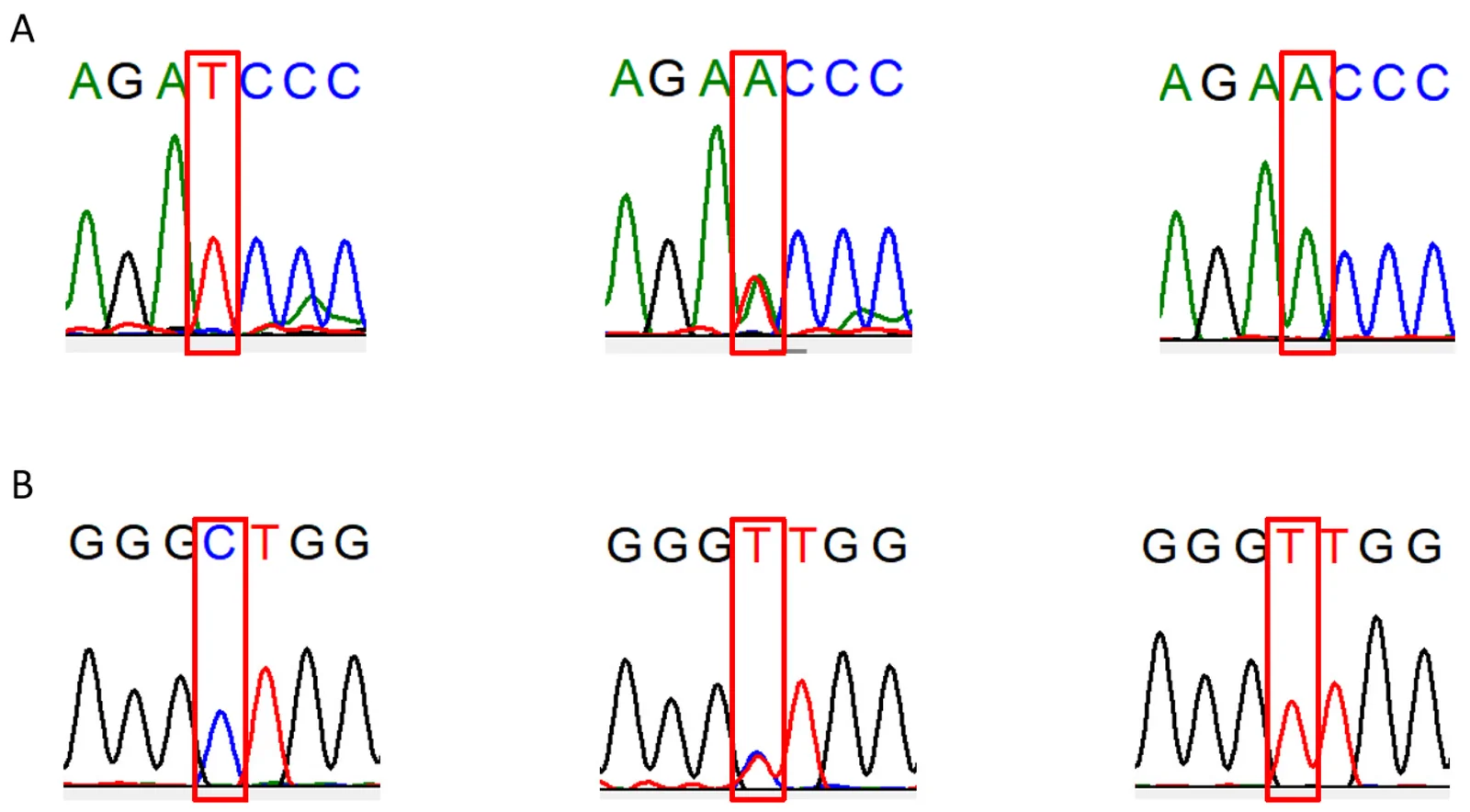
Sanger sequencing chromatograms of the two candidate SNP loci in Tianzhu white yak: (**A**) *CALCRL* gene chr 2:140178268 A > T; (**B**) *MPC1* gene chr 10:98419837 C > T.

PCR was used to amplify the target regions of *CALCRL* and *MPC1* genes in Tianzhu white yak. After PCR products of the expected sizes were confirmed by agarose gel electrophoresis, the products were subjected to Sanger sequencing. After sequence comparison and analysis, the sequencing chromatograms for each locus are shown in Figure 1, in which Figure 1A shows chr 2:140178268 A > T and Figure 1B shows chr 10: 98419837C > T. The genotype frequency, allele frequency and polymorphism information of two SNP loci of the *CALCRL* and *MPC1* genes are shown in Table 2. The results showed that three genotypes were identified at each SNP locus in the Tianzhu white yak population.

**Figure 2:**
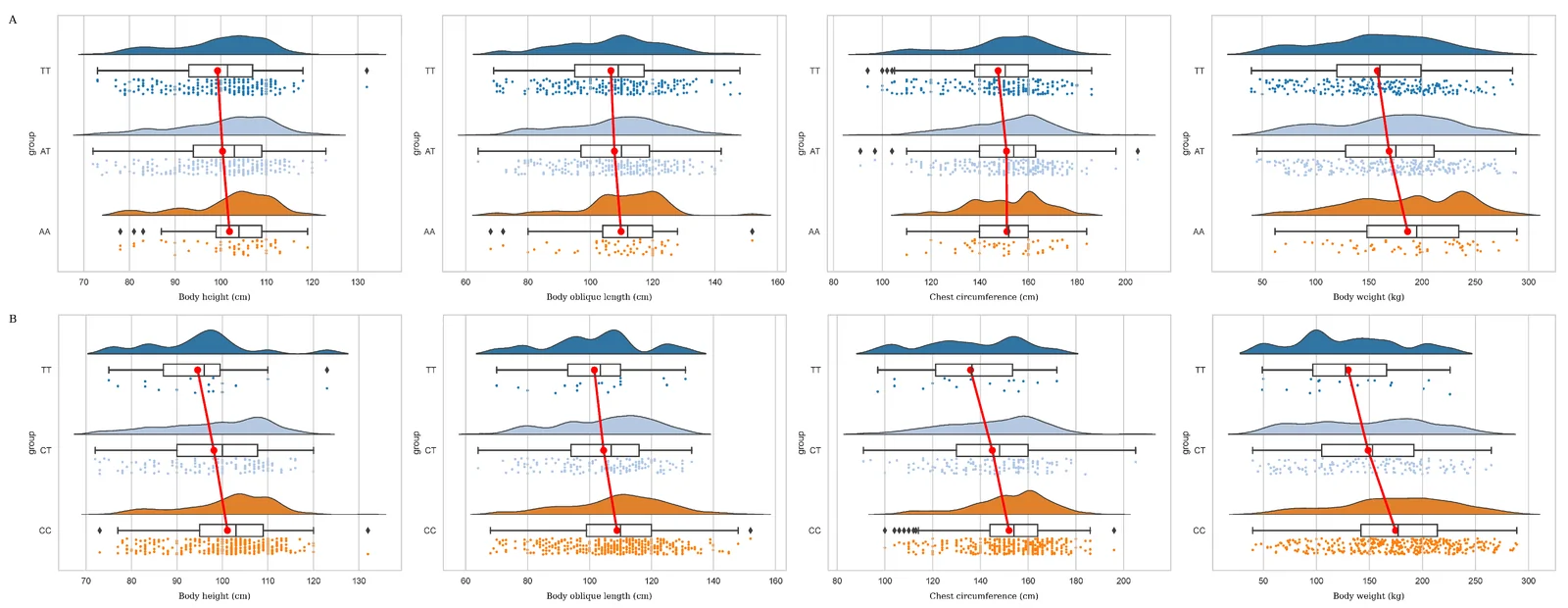
Phenotypic distributions of four body traits across genotypes at the two candidate SNP loci in Tianzhu white yak: (**A**) chr 2:140178268 A > T; (**B**) chr 10:98419837 C > T. The red line represents the average value of growth traits corresponding to the three genotypes. These three different colored points represent the distribution of data.

In order to directly explain the association between the SNP loci of the *CALCRL* and *MPC1* genes and growth traits, we analyzed the phenotypic distribution of the two identified loci based on genotype (Figure 2). The phenotypic distributions of BH, BOL, CC, and BW across genotype classes at the two loci are shown in Figure 2.

Among the genotype frequencies of the *CALCRL* gene chr 2:140178268 A > T and *MPC1* gene chr 10:98419837 C > T, the genotype frequencies of AT and CC are the highest, which are 0.478 and 0.707, respectively. This indicates that the AT genotype was the most frequent genotype at the chr 2:140178268 A > T site. At the chr 10:98419837 C > T locus, the CC genotype was the most frequent genotype. In chr 2:140178268 A > T and chr 10:98419837 C > T, the allele frequencies were higher for T and C, respectively, indicating that The T allele was more frequent at chr 2:140178268 A > T, whereas the C allele was more frequent at chr 10:98419837 C > T in chr 2:140178268 A > T; at the chr 10:98419837 C > T locus, the C allele was more frequent than the T allele. PIC of chr 2:140178268 A > T and chr 10:98419837 C > T are 0.350 and 0.236, respectively. Both SNP loci were consistent with Hardy–Weinberg equilibrium (*p* > 0.05).

### 3.2. Associations of SNP Genotypes with Body Weight and Body Measurements in Tianzhu White Yak

Based on the SNP genotyping data, the relationship between the chr 2:140178268 A > T locus of the *CALCRL* gene and the chr 10:98419837 C > T locus of the *MPC1* gene and different growth traits of Tianzhu white yak was analyzed. The results are shown in Table 3. After adjustment for age and sex, the genotype at chr 2:140178268 A > T was significantly associated with body weight, whereas no significant associations were detected with body height, body oblique length, or chest circumference. Among them, Individuals with AA and AT genotypes had significantly greater adjusted body weight than individuals with the TT genotype.

Genotype at chr 10:98419837 C > T was significantly associated with body height, chest circumference, and body weight, but was not significantly associated with body oblique length. Among them, the body height of individuals with the CC homozygous genotype was significantly higher than those with the TT genotype, but there was no significant relationship between them and the CT genotype. The chest circumference and weight of individuals with the CC homozygous genotype were significantly higher than those with the CT and TT genotypes.

## 4. Discussion

Body weight and body measurements in Tianzhu white yak are influenced by both genetic and environmental factors, including genetic background and feeding management [18,19,20]. Body weight and body measurements in yak are complex quantitative traits influenced by genetic background, age, sex, nutrition, and production environment. In recent years, an increasing number of SNPs associated with body-weight and body-size traits have been identified in different yak breeds and populations, including variants in *SIRT1*, *H-FABP*, *TXK*, and *PLCE1*, as well as multiple loci identified by genome-wide association studies [5,6,7,8,9]. These findings indicate that growth-related traits in yak have a polygenic basis and that the effects of candidate loci may vary among breeds, ages, and management environments. Therefore, it is important to carry out molecular genetic research on growth traits of yak and screen the key genetic loci affecting body height, weight and other traits.

The *CALCRL* gene chr 2:140178268 A > T locus was significantly associated with body weight, but not with body height, body oblique length and chest circumference. The body weight of individuals with AA and AT genotypes was significantly higher than that of individuals with the TT genotype, but there was no significant difference between AA and AT genotypes. A direct role for *CALCRL* in body-weight regulation in yak has not yet been established. The *CALCRL*-encoded receptor interacts with RAMPs and plays a central role in immune regulation, vasodilation and inflammatory response [21]. Changes in *CALCRL* expression may influence of *CALCRL* gene will affect animal cell proliferation, cardiovascular system development, hematopoiesis and immune stress regulation, which may indirectly affect animal growth [22,23]. These functions provide biological plausibility for a relationship with body-development phenotypes, but the connection remains indirect. The chr 2:140178268 A > T variant is located in a downstream non-coding region. Variants in downstream non-coding regions may potentially influence gene expression by affecting transcription termination, pre-mRNA processing, or transcription-factor binding [24,25]. However, these mechanisms were not examined in the present study. Consequently, the observed association may reflect a regulatory effect of the locus or linkage disequilibrium with another functional variant.

The chr 10:98419837 C > T locus of the *MPC1* gene was significantly associated with body height, chest circumference and body weight, but not with body oblique length. The body height of individuals with the CC genotype was significantly higher than that of individuals with the TT genotype, and their chest circumference and weight were significantly higher than those of individuals with the CT and TT genotypes. These results suggest that the *MPC1* genome region may be related to the variation in several body-size traits in the Tianzhu White Yak population.

*MPC1* plays a role in cell energy metabolism, biosynthesis, embryo and organ development, immune regulation and systemic homeostasis maintenance [14,17]. *MPC1* and *MPC2* form heterodimers, which are responsible for transporting pyruvate produced in the cytoplasm into mitochondria [14]. *MPC1* dependent pyruvate transport plays an important role in cell energy metabolism by participating in the process of pyruvate entering mitochondria and connecting glycolysis and mitochondrial oxidative metabolism. *MPC1* has also been implicated in skeletal-muscle metabolic regulation and muscle-fiber development in other livestock species [15]. However, the present association should not be interpreted as evidence that chr 10:98419837 C > T directly regulates these phenotypes. Like the *CALCRL* linked SNP, this locus is located in a downstream non-coding region. No gene expression, transcriptional regulation, or cellular functional experiments were conducted in the present study. The SNP may therefore be a regulatory variant, or it may simply be in linkage disequilibrium with an unidentified causal mutation.

The genotype distribution at the *MPC1* linked locus also warrants caution. Only 18 animals carried the TT genotype, compared with 139 CT and 379 CC individuals. Although the association model adjusted for age and sex, estimates for the small TT group are less precise, as reflected by its larger standard errors. The phenotypic differences involving this genotype should therefore be interpreted cautiously and require confirmation in larger independent populations.

The practical significance of body-weight and body-size associations should also be considered within the production environment of Tianzhu white yak. Yaks are multipurpose livestock adapted to cold, hypoxic, and seasonally nutrient-limited plateau conditions. Greater mature body size may be advantageous for meat production under some circumstances, but it may also increase maintenance nutrient requirements. Conversely, relatively small body size could potentially contribute to the ability to maintain body condition when forage availability is limited. Therefore, a genotype associated with greater body weight should not automatically be interpreted as universally superior. Future breeding evaluations should consider possible relationships or trade-offs among body size, nutritional requirements, reproductive performance, health, environmental adaptation, milk production, fiber production, and other economically important traits.

The two SNP loci identified in the present study should not be interpreted as being superior to previously reported molecular markers associated with growth traits in yak. Their main significance lies in further expanding the current set of candidate markers for growth traits in Tianzhu white yak. At present, a meaningful prioritization framework among different candidate loci cannot be established solely on the basis of statistical significance observed in individual studies. Future prioritization of candidate markers should comprehensively consider replication in independent populations, consistency in the direction and magnitude of genetic effects, allele frequencies, associations with multiple economically important traits, functional annotation results, and other relevant experimental evidence.

Overall, the results of this study support that the *CALCRL* gene Chr 2:140178268 A > T and *MPC1* gene Chr 10:98419837 C > T are candidate genetic loci related to the body weight or body shape differences in the Tianzhu White Yak population in this study. However, the present study demonstrates statistical associations rather than causal effects. Independent population validation and functional characterization are required before these loci can be considered for marker-assisted selection.

## 5. Conclusions

After adjustment for age and sex, the *CALCRL* gene chr 2:140178268 A > T locus was significantly associated with the body weight of Tianzhu white yak, but not with body height, body oblique length and chest circumference. The chr 10:98419837 C > T locus of the *MPC1* gene was significantly associated with body height, chest circumference and weight, but not with body oblique length.

These findings identify the two loci as candidates for further investigation of body-weight and body-size variation in Tianzhu white yak. However, both SNPs are located in downstream non-coding regions, and their functional consequences remain unknown. Validation in independent yak populations, together with functional studies and evaluation of other economically and environmentally important traits, will be required before their potential application in marker-assisted breeding can be established.

## Figures and Tables

**Table 1:** Primer information for the two SNP loci.

SNP	Primer Sequences (5′-3′)	Product Size (bp)	Annealing Temperature (°C)
chr 2:140178268 A > T	F: CCATCTCCCCAAACTCCCTTC	304	59
	R: GTGTCTTACTGTGTGTGACCCT		
chr 10:98419837 C > T	F: GGAGGAGATCAAGCGCCATC	257	59
	R: GCAAAGGGCTGGACGGATT		

**Table 2:** Variation information and diversity parameters for the *CALCRL* gene (chr 2:140178268 A > T) and the *MPC1* gene (chr 10:98419837 C > T).

SNP	Genotype	Number	Frequency	Allele	Frequency	He	Ne	PIC	HWE *p*-Value
chr 2:140178268 A > T	AA	58	0.108	A	0.347	0.453	1.829	0.350	0.212
	AT	256	0.478	T	0.653				
	TT	222	0.414						
chr 10:98419837 C > T	CC	379	0.707	C	0.837	0.274	1.377	0.236	0.239
	CT	139	0.259	T	0.163				
	TT	18	0.034						

He, expected heterozygosity; Ne, effective number of alleles; PIC, polymorphism information content; HWE, Hardy–Weinberg equilibrium.

**Table 3:** Associations of *CALCRL* and *MPC1* SNP genotypes with body weight and body measurements in Tianzhu white yak.

**SNPs chr 2:140178268 A > T**					
Genotype	*n*	Body height (cm)	Body oblique length (cm)	Chest circumference (cm)	Body weight (kg)
AA	58	101.08 ± 0.87	108.90 ± 1.35	148.64 ± 1.80	180.94 ± 4.19 ^a^
AT	256	101.04 ± 0.59	109.29 ± 0.91	150.58 ± 1.21	172.43 ± 2.83 ^a^
TT	222	100.38 ± 0.62	108.71 ± 0.96	148.99 ± 1.28	165.64 ± 2.99 ^b^
*p*-Value		0.470	0.223	0.304	0.001
**SNPs chr 10:98419837 C > T**					
Genotype	*n*	Body height (cm)	Body oblique length (cm)	Chest circumference (cm)	Body weight (kg)
CC	379	101.44 ± 0.55 ^a^	109.70 ± 0.86	151.35 ± 1.13 ^a^	177.17 ± 2.64 ^a^
CT	139	100.30 ± 0.65 ^ab^	108.34 ± 1.02	147.99 ± 1.35 ^b^	164.15 ± 3.14 ^b^
TT	18	96.85 ± 1.44 ^b^	105.29 ± 2.24	140.77 ± 2.97 ^b^	148.52 ± 6.93 ^b^
*p*-Value		0.002	0.072	<0.001	<0.001

Values are estimated marginal means ± standard errors (SE), adjusted for age and sex. Within each SNP locus and trait, means with different superscript letters differ significantly according to Bonferroni-adjusted pairwise comparisons (*p* < 0.05). *p*-values represent the overall genotype effects obtained from the general linear model.

## Data Availability

The genotypic data used and/or analyzed during the current study are available from the corresponding author on reasonable request.

## Abbreviations

The following abbreviations are used in this manuscript:

SNP	Single nucleotide polymorphism
BH	Body height
BOL	Body oblique length
CC	Chest circumference
BW	Body weight
He	Expected heterozygosity
Ne	Effective number of alleles
PIC	Polymorphism information content
